# Supplementary material for: Occurrence of Capnophilic Lactic Fermentation in the Hyperthermophilic Anaerobic Bacterium Thermotoga sp. Strain RQ7
Source: Int J Mol Sci. 2022 Oct 10;23(19):12049. doi: 10.3390/ijms231912049 (PMC9570489; doi:10.3390/ijms231912049)
Supplement: Supplementary file 1 [file ijms-23-12049-s001.zip › ijms-1873460-supplementary.pdf]

# Supplementary Materials

## Assessment of Capnophilic Lactic Fermentation (CLF) pathway in thermophilic anaerobic bacteria *Thermotoga neapolitana, Thermotoga maritima* and *RQ7*

**Nunzia Esercizio[1], Mariamichela Lanzilli[1], Simone Landi [1-2], Lucio Caso[1], Zhaohui Xu [3], Genoveffa Nuzzo[1], Carmela Gallo[1], Emiliano Manzo[1], Sergio Esposito[2], Angelo Fontana[1,2] and Giuliana d'Ippolito[1,*]**

[1] Institute of Biomolecular Chemistry (ICB), CNR, Via Campi Flegrei 34, 80078 Pozzuoli (Napoli), Italy
[2] Department of Biology, University of Naples "Federico II", Via Cinthia, I-80126 Napoli, Italy
[3] Department of Biological Sciences, Bowling Green State University, Bowling Green, OH 43403, USA
[*] Correspondence: gdippolito@icb.cnr.it , +39 0818675096

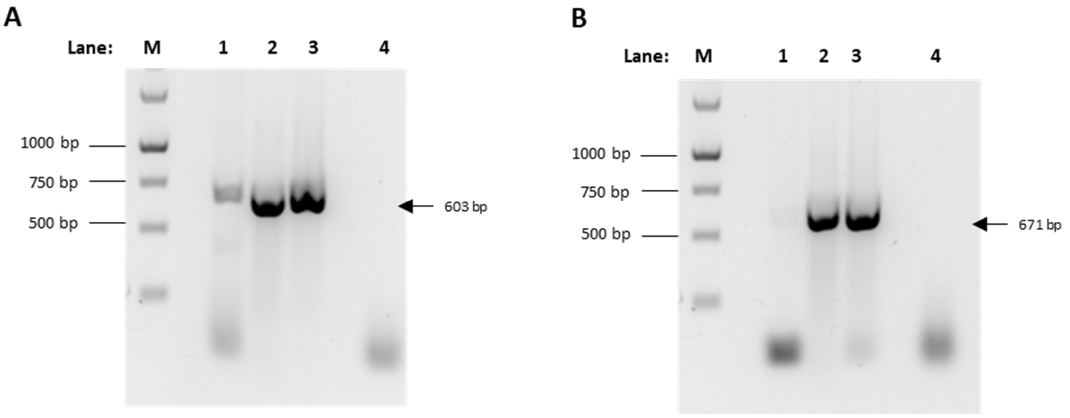


**Figure S1:** PCR results for the presence of F-type (**A**) and V-type (**B**) ATPases on the genomic DNA of *T.nea, T.mar* and *T.rq7*. In sequence: M, marker 1Kb; Lane 1, *T.maritima*; Lane 2, *T.neapolitana*; Lane 3, *T.rq7*; Lane 4, negative control. Fragments of 603 bp and 671 bp respectively are expected.

**Table S1:** Bioinformatic analysis of V-ATPases in *Thermotogacea* family. For each subunit, species and percentage of identity in relation to *T.nea* are indicated.

| **V-ATPase subunit in *T.nea*** | ***Trq7*** | **% ID** | ***P.hyp*** | **%ID** |
|---|---|---|---|---|
| subunit A-atpA | TRQ7_06035 | 100 | AJ81_03190 | 46.37 |
| subunit B-atpB | TRQ7_06040 | 100 | AJ81_03195 | 51.92 |
| subunit D-atpD | TRQ7_06045 | 100 | AJ81_03200 | 31.01 |
| subunit E-atpE | TRQ7_06030 | 99.8 | AJ81_03175 | 27.51 |
| subunit F-atpF | TRQ7_06020 | 100 | AJ81_03165/ AJ81_03170 | 23.26/ 23.49 |
| subunit G-atpG | TRQ7_06025 | 100 | AJ81_03165/ AJ81_03170 | 26.79/ 23.05 |

**Table S2:** Bioinformatic analysis of F-ATPases in *Thermotogacea* family. For each subunit, species and percentage of identity in relation to *T.nea* are indicated.

| ***T.nea* subunits** | **T*mar*** | ***% ID*** | ***Trq7*** | ***%ID*** | ***Tnap*** | ***%ID*** | ***Tpet*** | ***%ID*** | ***P.lett*** | ***%ID*** | ***P.ther*** |
|---|---|---|---|---|---|---|---|---|---|---|---|
| subunit A-atpB | TM_1616 | 76.52 | TRQ7_05640 | 100 | Tnap_1191 | 75.44 | Tpet_1175 | 76.39 | Tlet_0160 | 51.4 | Theth_0 |
| subunit B-atpF | TM_1614 | 75.75 | TRQ7_05650 | 91.92 | Tnap_1193 | 76.97 | Tpet_1177 | 75.75 | Tlet_0162 | 43.96 | Theth_0 |
| subunit C-atpE | TM_1615 | 86.43 | TRQ7_05645 | 98.83 | Tnap_1192 | 86.04 | Tpet_1176 | 86.43 | Tlet_0161 | 50.19 | Theth_02<br>Theth_0 |
| subunit Delta-atpH | TM_1613 | 71.37 | TRQ7_05655 | 99.82 | Tnap_1194 | 69.38 | Tpet_1178 | 71.37 | Tlet_0163 | 39.89 | Theth_0 |
| ubunit Epsilon-atpC | TM_1609 | 77.47 | TRQ7_05675 | 98.15 | Tnap_1198 | 77.47 | Tpet_1182 | 77.47 | Tlet_0167 | 33.02 | Theth_0 |
| ubunit Gamma-atpG | TM_1611 | 76.27 | TRQ7_05665 | 100 | Tnap_1196 | 75.79 | Tpet_1180 | 76.58 | Tlet_0165 | 42.68 | Theth_0 |
| subunit Alpha-atpA | TM_1612 | 82.87 | TRQ7_05660 | 99.88 | Tnap_1195 | 82.87 | Tpet_1179 | 82.93 | Tlet_0164 | 64.02 | Theth_0 |
| subunit I-atpI | - | - | TRQ7_05635 | 100 | - | - | - | - | - | - | - |
| subunit Beta- atpD | TM_1610 | 81.31 | TRQ7_05670 | 97.87 | Tnap_1197 | 81.31 | Tpet_1181 | 81.31 | Tlet_0166 | 67.23 | Theth_0 |

**Table S3:** List of primers used for PCR and Real-time PCR analysis on *T.nea, T.mar* and *T.rq7*.

| Primer | Sequence | Description |
|---|---|---|
| PCR-VATPa Fw | AGAGTAAGGGGAGGAGACGT | alfa subunit of V-type ATPase for classic PCR |
| PCR-VATPa Rv | ACGGGCATTTCTTCCAGTCT | |
| PCR-FATPb Fw | GACAGATGAACGAACCACCG | beta subunit of F-type ATPase for classic PCR |
| PCR-FATPb Rv | CAACGTGGAAAGGCTGACTC | |
| RT-VATPa Fw | AGATGGGCAGAGGCATTGAG | alfa subunit of V-type ATPase for real time PCR |
| RT-VATPa Rv | ACTGAGCGATTCTGGTAGCG | |
| RT-VATPb Fw | AGTGCGTTCTATCGCTTCCA | beta subunit of V-type ATPase for real time PCR |
| RT-VATPb Rv | AAGGTGCTCGAATTCTTCGC | |
| RT-FATPb Fw | AACCACCTTCGCTCACCTG | beta subunit of F-type ATPase for real time PCR |
| RT-FATPb Rv | TCGAAGAAGAATCGAGCGGG | |
